# Supplementary material for: Divergent iron regulatory states contribute to heterogeneity in breast cancer aggressiveness
Source: iScience. 2024 Aug 3;27(9):110661. doi: 10.1016/j.isci.2024.110661 (PMC11387597; doi:10.1016/j.isci.2024.110661)
Supplement: Document S1. Figures S1–S6 [file mmc1.pdf]

iScience, Volume *27*

Supplemental information

Divergent iron regulatory states contribute to heterogeneity in breast cancer aggressiveness

William D. Leineweber, Maya Z. Rowell, Sural K. Ranamukhaarachchi, Alyssa Walker, Yajuan Li, Jorge Villazon, Aida Mestre-Farrera, Zhimin Hu, Jing Yang, Lingyan Shi, and Stephanie I. Fraley

Figure S1

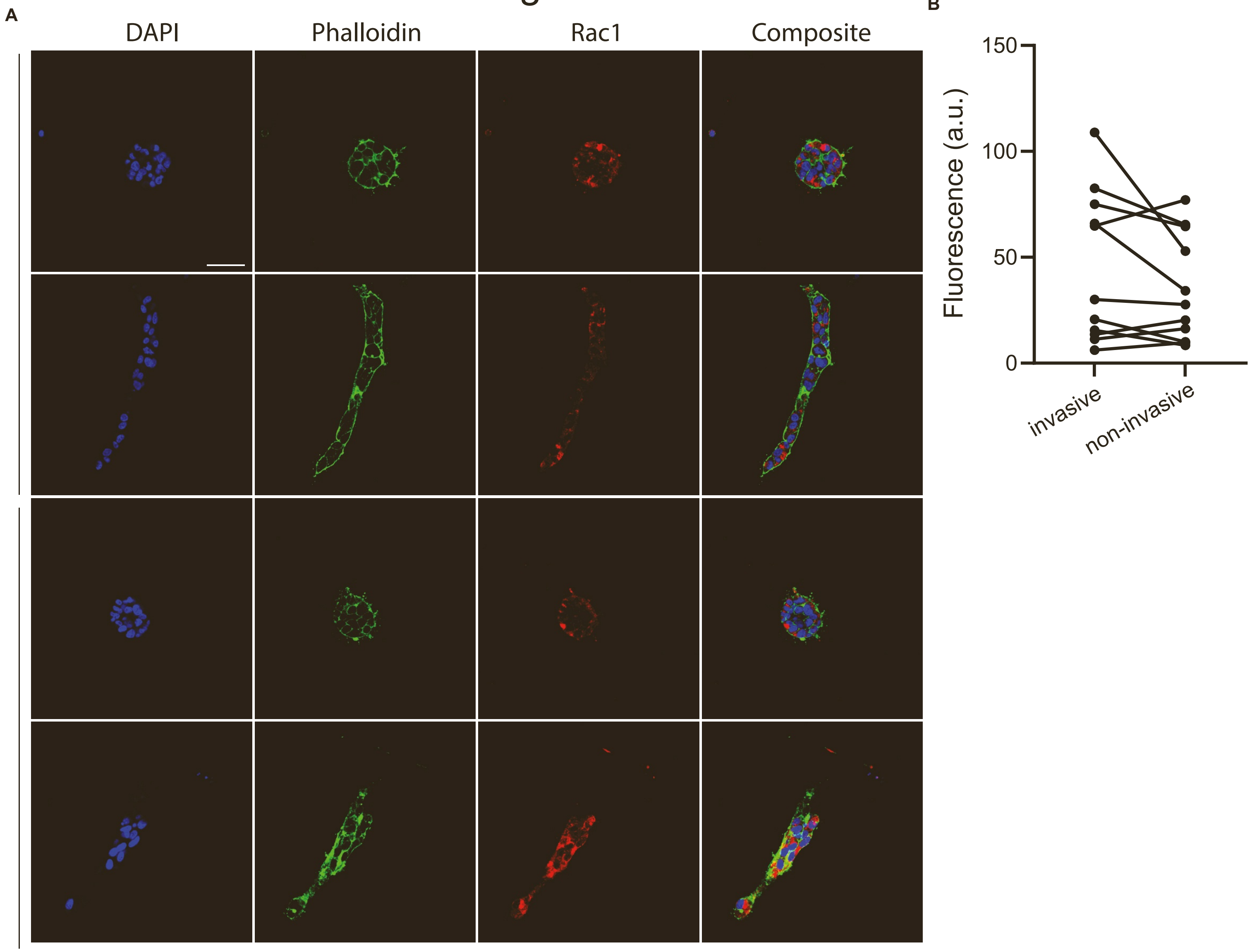

Figure S1: Immunostaining of RAC1 shows no quantitative differences between invasive and non-invasive phenotypes, Related to Figure 1.
MDA-MB-231 cells cultured in 6mg/ml Col1 for seven days were fixed and stained to show RAC1 expression levels. (A) Representative micrographs from confocal microscopy show the nuclei, actin, and RAC1 protein levels. Scale bar = 200µm. (B) The average fluorescence intensity was measured within each multicellular structure and no significant difference between the phenotypes was observed. Pairwise comparisons of invasive and non-invasive structures from within the same collagen gels were used to account for fluctuations in fluorescence intensity arising from staining or imaging factors.

Fig. S2

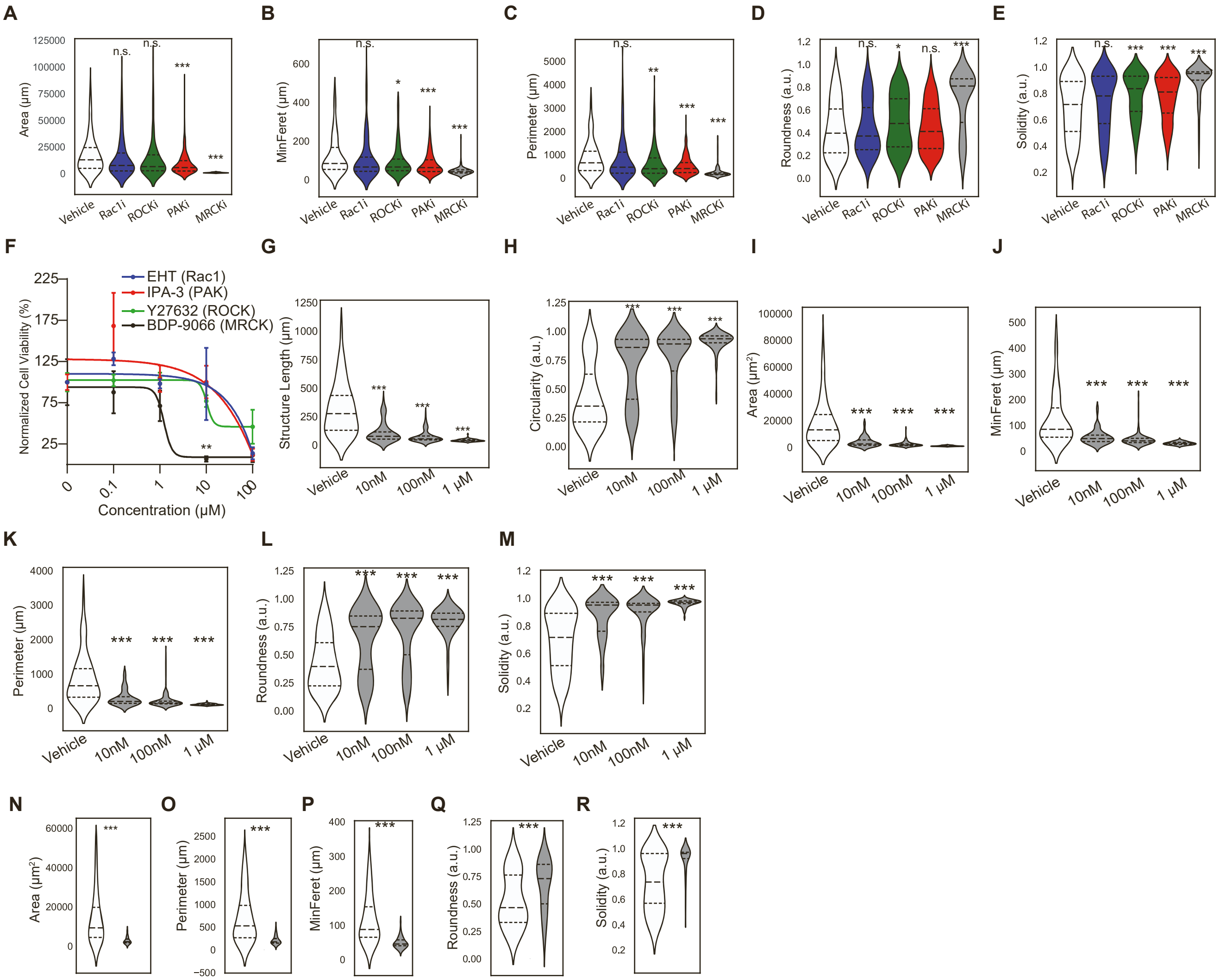

Figure S2: Effects of cytoskeletal inhibitors on collective migration phenotypes and viability, Related to Figure 1.
(A-E) Shape descriptors of MDAs embedded in HD Col1 for seven days after initial 24hr treatment with cytoskeletal inhibitors. (F) MTS assay of MDAs embedded in HD Col1 for seven days after initial 24hr treatment with cytoskeletal inhibitors. (G-M) Shape descriptors of MDAs embedded in HD Col1 for seven days after initial 24hr treatment with different concentrations of MRCK inhibitors. (N-R) Shape descriptors of 4T1s embedded in HD Col1 for seven days after initial 24hr treatment with MRCK inhibitor BDP-9066 at 1μM.

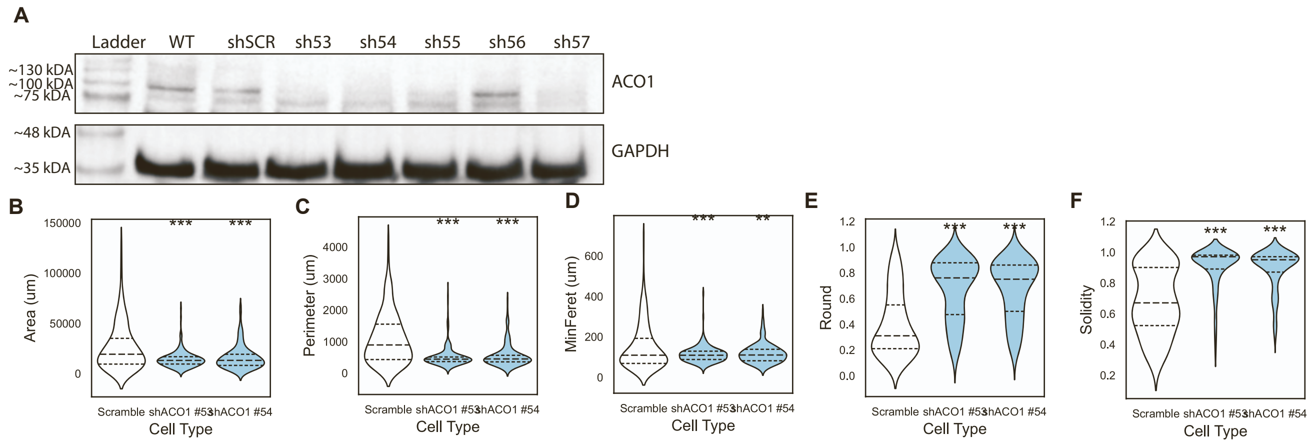

Figure S3: shACO1 shape quantification, Related to Figure 2.
(A) Expanded Western blot validating the shRNA knockdowns of ACO1 protein levels. (B-F) Shape descriptors of shACO1 MDAs embedded in HD Col1 for seven days.

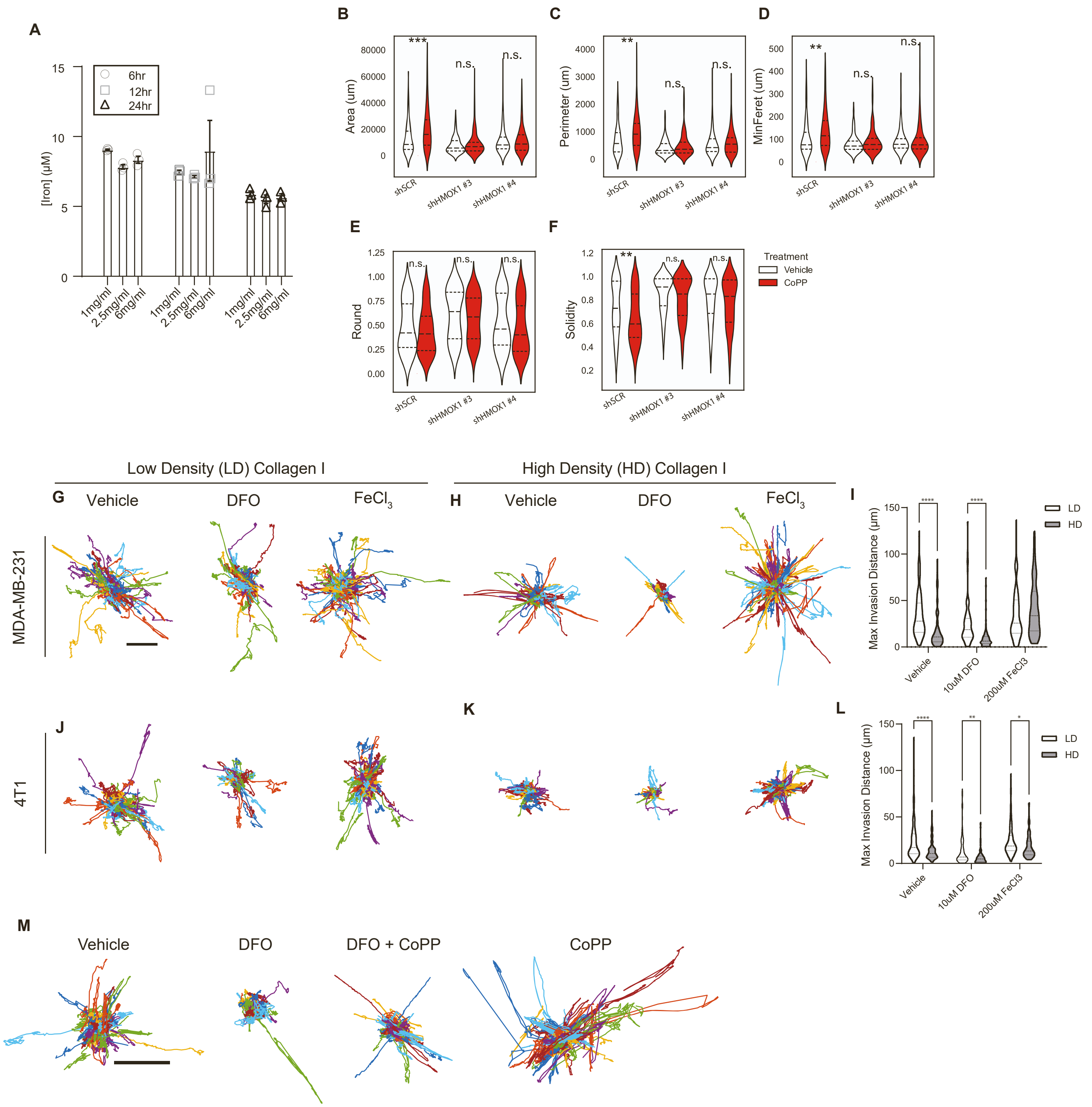

Figure S4: Perturbing extracellular and intracellular iron levels alters single cell and collective invasion, Related to Figure 4.

(A) Chelatable iron levels in DMEM following incubation with collagen I matrices with varying concentrations. (B-F) Shape descriptors of MDAs embedded in HD Col1 for seven days with media supplemented with 10µM CoPP. (G-I) Trajectories of MDAs embedded in LD (G) or HD (H) matrices and the (I) associated max invasion distances. (J-L) Trajectories of MDAs embedded in (J) LD or (K) HD matrices and the (L) associated max invasion distances. (M) Trajectories of MDAs in HD matrices treated with 0.1% DMSO, 10µM DFO, 10µM DFO + 100µM CoPP, or 100µM CoPP.

A

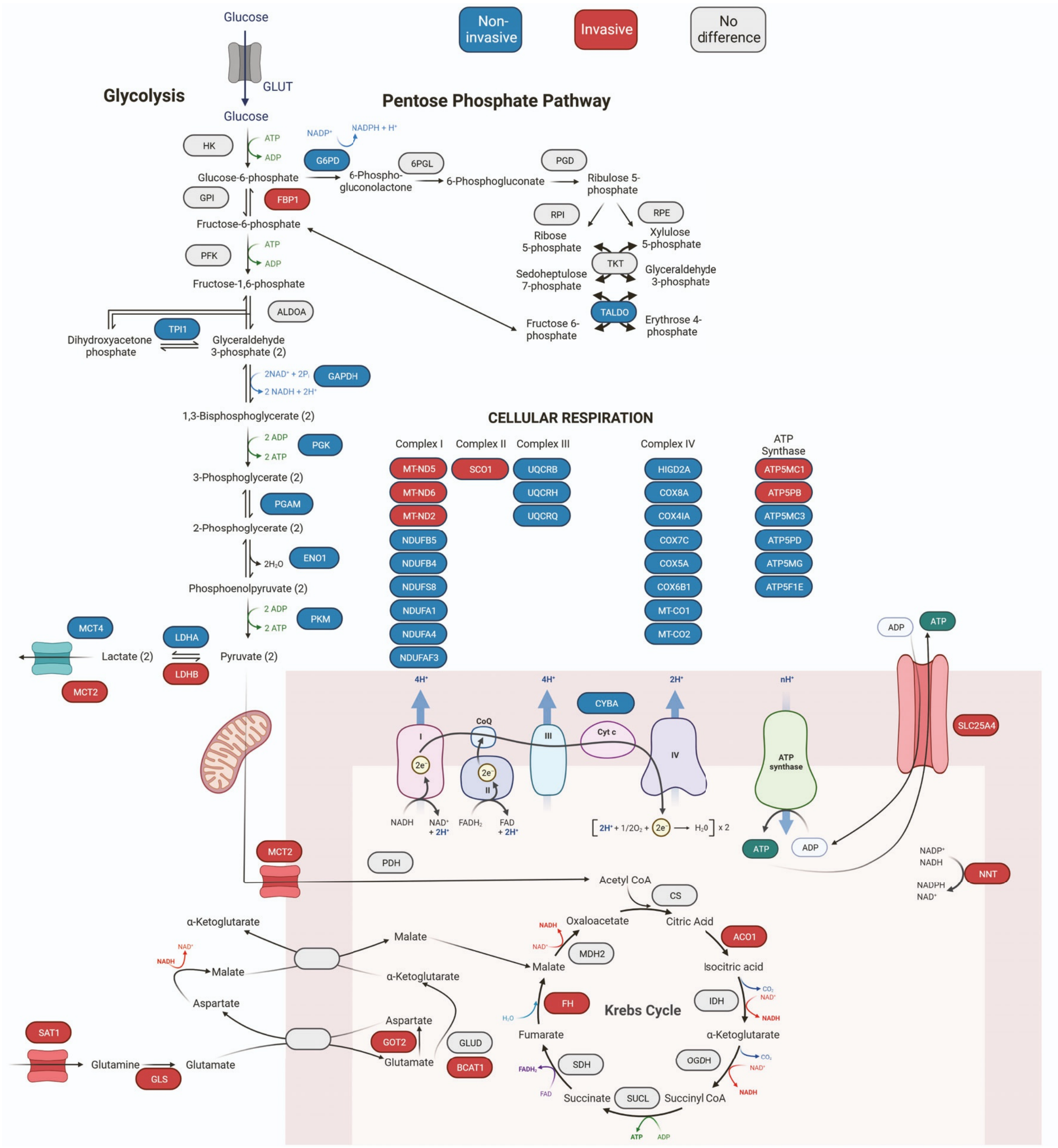

Figure S5: Differentially expressed genes mapped to glucose metabolism, Related to Figure 6.
(A) Essential proteins involved in glucose metabolism through glycolysis, the TCA cycle, and oxidative phosphorylation. Shunting from glycolysis to the pentose phosphate pathway is also shown, as are anapleirotic sources of the TCA cycle.

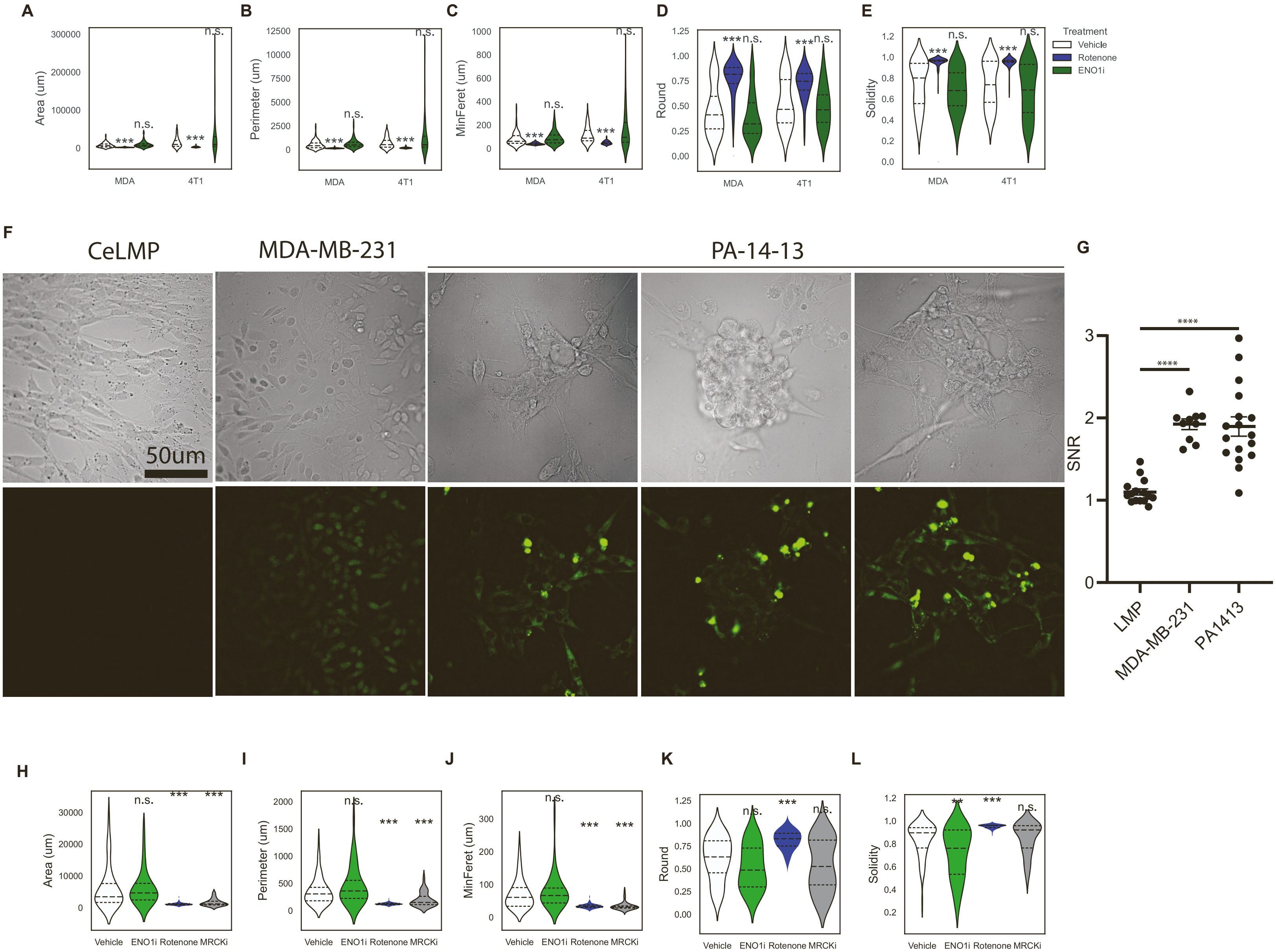

Figure S6: Metabolic inhibitors extended shape descriptors and PDO model validation, Related to Figure 7.
(A-E) Shape descriptors of MDAs and 4T1s embedded in HD Col1 for seven days after initial 24hr treatment with inhibitors targeting Complex I of the electron transport chain and glycolytic enzyme ENO1. (F) Cells from the PDOs after excision from mouse xenografts were validated to be human via staining with a human-specific antibody. While mouse CeLMP cells have no signal, the PA-14-13 model shows staining at a similar level as MDAs. (G) Quantification of the signal-to-noise ratio (SNR).
(H-L) Shape descriptors of the PA-14-13 PDO model after treatment with inhibitors targeting Complex I of the electron transport chain and glycolytic enzyme ENO1.
